# Negative Evidence for a Functional Role of Neuronal DNMT3a in Persistent Pain

**DOI:** 10.3389/fnmol.2018.00332

**Published:** 2018-09-12

**Authors:** Jessica Saunders, Zoe Hore, Clive Gentry, Stephen McMahon, Franziska Denk

**Affiliations:** Wolfson Centre for Age-Related Diseases, King’s College London, London, United Kingdom

**Keywords:** epigenetics, pain, DNMT, DNA methyltransferase, sensory neurons, knockout mice

## Abstract

Traditionally, neuroscience has had to rely on mixed tissue analysis to examine transcriptional and epigenetic changes in the context of nervous system function or pathology. However, particularly when studying chronic pain conditions, this approach can be flawed, since it neglects to take into account the shifting contribution of different cell types across experimental conditions. Here, we demonstrate this using the example of DNA methyltransferases (DNMTs) – a group of epigenetic modifiers consisting of Dnmt1, Dnmt3a, and Dnmt3b in mammalian cells. We used sensory neuron-specific knockout mice for Dnmt3a/3b as well as pharmacological blockade of Dnmt1 to study their role in nociception. In contrast to previous analyses on whole tissue, we find that Dnmt3a and 3b protein is not expressed in adult DRG neurons, that none of the DNA methyltransferases are regulated with injury and that interfering with their function has no effect on nociception. Our results therefore currently do not support a role for neuronal DNA methyltransferases in pain processing in adult animals.

## Introduction

Persistent pain conditions are common within the general population, affecting about one in five individuals during the course of their lives (Breivik et al., 2006). Particularly devastating for patients is chronic pain arising as a result of direct injury or disease of the nervous system, known as neuropathic pain. It can occur in many disease contexts, for instance diabetes, shingles, or stroke. Patients can experience a variety of painful symptoms, e.g., spontaneous burning or shooting pain and evoked pain in response to non-noxious stimuli. Regardless of disease etiology, the disorder is thought to be characterized by long-lasting hypersensitivity in the nervous system, not only in peripheral sensory neurons, but also in spinal cord and cortical top-down modulatory circuitry (Baron et al., 2010).

It is unclear why this hypersensitivity should remain established over such long periods of time. One way in which cell function can be impacted over long periods of time is through epigenetics (Denk and McMahon, 2017). Indeed, the role of epigenetic mechanisms is increasingly being studied in the pain field (Denk et al., 2016; Denk and McMahon, 2017; Niederberger et al., 2017), as well as in neuroscience in general (Akbarian et al., 2015; Gray et al., 2015; Mo et al., 2015; Halder et al., 2016; Hannon et al., 2016).

Methylation of cytosines within the DNA sequence is one such epigenetic mechanism. In mammalian cell types, it most frequently occurs at cytosine–guanine (CG) sites, although in neurons specifically, up to a fourth of methylated cytosines occur in a non-CG context (Lister et al., 2013). Methyl groups are added by enzymes known as DNA methyltransferases (DNMTs). There are three catalytically active DNMTs in mammals, all of them crucial for normal development and survival: Dnmt1, Dnmt3a, and Dnmt3b. Dnmt1 is thought to primarily copy existing DNA methylation patterns upon replication, while Dnmt3a and Dnmt3b are *de novo* methyltransferases, capable of modifying naked CG sites. After development, Dnmt3a continues to be expressed in post-mitotic neurons in several areas of the brain (Feng et al., 2005), and studies in knockout mice have suggested that it plays a role in a variety of neuronal functions, such as motor coordination (Nguyen et al., 2007), olfaction (Colquitt et al., 2014) and synaptic plasticity in the context of memory (Feng et al., 2010; Morris et al., 2014) and reward (LaPlant et al., 2010). Thus, it seemed reasonable to hypothesize that DNMTs might also be relevant for processing of sensory stimuli. Indeed, last year, two back-to-back publications from the same group suggested a role for Dnmt3a in nociceptive neuron function and pain processing after nerve injury (Sun et al., 2017; Zhao et al., 2017).

However, here we present evidence that runs counter to these findings. In contrast to previous work, we use cell-type specific data, pharmacological tools and a sensory neuron-specific Dnmt3a/3b knockout mouse to demonstrate that DNMTs are unlikely to play a role in sensory neurons in adult animals. Most strikingly, our data indicate that Dnmt3a and 3b proteins are not expressed in adult DRG neurons and are not upregulated with injury. In line with this, their knockout had no measurable effect on nociception.

## Materials and Methods

### Animals

For the experiment in **Figure 6**, C57BL/6J male mice were purchased from Harlan at 8 weeks of age and acclimatized for 1 week before behavioral testing.

For all other experiments, age-matched cohorts of mice were bred from Dnmt3a/Dnmt3b double floxed mice (Dnmt3a^fl/fl^/Dnmt3b^fl/fl^) (Dodge et al., 2005) crossed with an inducible sensory neuron-specific Cre line (AdvCre^ERT2^) (Lau et al., 2011). AdvCre^ERT2^ positive and negative littermates were generated through breeding from one hemizygous BAC+ and one BAC negative parent. The resulting line was maintained on a 129Sv hybrid background, and consequently showed higher baseline sensitivity to nociceptive stimuli than C57BL/6J mice, as described by Mogil et al. (1999a,b).

Mice were housed in standard open-topped cages in groups of five maximum at 12 h light–dark cycle. All experiments described were carried out in accordance with the United Kingdom Home Office Legislation (Scientific Procedures Act, 1986) and were approved by the Home Office to be carried out at King’s College London under project license 70/8015.

### Drugs

Tamoxifen (T5648, Sigma) was used to induce deletion of Dnmt3a and Dnmt3b in sensory and sympathetic neurons in a time-dependent manner. 1 g of tamoxifen was weighed out and vortexed in 2 ml 100% EtOH before being dissolved in 23 ml sun flower or wheat germ oil through vigorous shaking for 30 min (final concentration: 7.8 mg/ml). The compound was delivered to mice three times in 1 week, each time injected *i.p.* at 75 mg/kg.

RG108 was obtained from Abcam (ab141013) and delivered at 15 μg intrathecally in 9% DMSO. Vehicle controls were injected with 9% DMSO only. For the CFA experiment, RG108 was injected 30 min before and 47 h after CFA administration. In the partial sciatic nerve ligation model, RG108 was injected on three consecutive days, starting 30 min before surgery. An additional “booster” dose was delivered 8 days after surgery.

### Complete Freund’s Adjuvant Model (CFA)

After obtaining baseline von Frey thresholds, C57BL/6J mice were pseudo-randomized into groups and treated with RG108 or vehicle (*n* = 8) 30 min before an intraplantar CFA injection (F5881, Sigma, 20 μl per paw). A second RG108 injection was given 47 h after CFA. Behavior was assessed with von Frey hairs using the Dixon up–down method (Chaplan et al., 1994) at 5, 22, 48, and 72 h after CFA by an experimenter blind to treatment group. Each time, the mice were allocated to different von Frey boxes at random. Three hours after the last behavioral time point, the animals were overdosed with pentobarbital and perfused with PBS. Lumbar DRG (L3-L5) were removed, snap frozen in liquid nitrogen and stored at −80°C for subsequent ELISA.

### Partial Sciatic Nerve Ligation Model

A cohort of male AdvCre^ERT2^-Dnmt3a^fl/fl^/Dnmt3b^fl/fl^ and their age-matched littermate controls (Dnmt3a^fl/fl^/Dnmt3b^fl/fl^, *n* = 9) were treated with tamoxifen as described above and, 18 days later, subjected to partial sciatic nerve ligation under isofluorane anesthesia. Briefly, after administration of an analgesic (0.05 mg/kg buprenorphine), the sciatic nerve was exposed through blunt dissection and tightly ligated with a 5.0 suture. The wound was stapled shut, and the mice left to recover.

In addition to the genetic manipulation of Dnmt3a and Dnmt3b, mice were also intermittently treated with intrathecal RG108 to insure against any compensatory effects of Dnmt1 (see above and **Supplementary Table 1** for details).

Mechanical and thermal sensitivity was assessed using von Frey and hot plate tests at baseline before, as well as at varying intervals after surgery (see **Supplementary Tables 2**, **3** for details). All testing was carried out by an experimenter blind to genotype and RG108 treatment group, and on different testing days, mice were allocated to different von Frey boxes at random.

### Protein Extraction and Western Blotting

DRG, spinal cord and brain were extracted from AdvCre^ERT2^-Dnmt3a^fl/fl^/Dnmt3b^fl/fl^ transgenic mice and their littermate controls (*n* = 3) either with or without prior PBS perfusion. Depending on the experiment, extractions were performed 1–4 weeks after tamoxifen injections.

For protein extraction, the tissue was shaken for 2 h at room temperature in 0.2% SDS and protease inhibitors in ultrapure water. The remaining extracellular matrix was spun down and the supernatant removed for Nanodrop quantification before Western blotting.

Western blots were carried out according to standard protocols, with samples being run on 4–12% Bis-Tris gels and being transferred onto PVDF membranes. The following antibodies were used: Dnmt3a (mouse, 1:250, Enzo, ALX-804-370-C100) and Gapdh (rabbit, 1:10000, Sigma, G9545) or α-tubulin (mouse, 1:1000, Sigma, T9026) as loading controls.

### 5-mC ELISA

DNA was extracted from L3-L5 DRG using a Qiagen DNeasy Blood & Tissue Kit (69504) following manufacturer’s protocols. Once purified, the DNA was diluted to 8 ng/μl in 5-mC buffer delivered with the 5-mC DNA ELISA Kit (Zymo Research, D5325). Kit instructions were then followed. Briefly, 100 ng of DNA and methylation standards (0–100% methylated 5-mC) were used to coat a PCR plate. The samples were washed, before blocking in 200 μl 5-mC ELISA buffer for 30 min and incubation with primary (5-mC) and secondary antibodies for 1 h. After further washes, the HRP signal was developed for 30 min at room temperature and read at 420 nM on a plate reader.

### RNA Extraction and qRT-PCR

**FIGURE 1:**
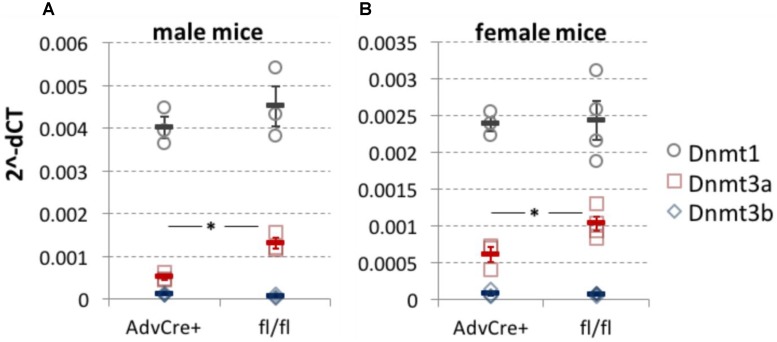
Dnmt3a knockdown after tamoxifen administration to AdvCre^ERT2^-Dnmt3a^fl/fl^/Dnmt3b^fl/fl^ mice. DNMT expression measured by qRT-PCR in whole L3-L5 DRG taken from sensory neuron specific Dnmt3a and Dnmt3b knockout mice. While Dnmt3b was absent, Dnmt3a knockout was significant in both male (**A**, two-tailed independent samples *t*-test, *p* = 0.01, *n* = 3) and female mice (**B**, two-tailed independent samples *t*-test, *p* = 0.03, *n* = 3 and 4). There appeared to be no compensatory shift in Dnmt1 expression as a result of the genetic manipulation. Values are expressed as 2^−ΔCT^ with respect to three housekeeping genes. Each data point (circles for Dnmt1, squares for Dnmt3a and diamonds for Dnmt3b) represents one biological replicate. Means and their standard errors are also displayed.

After the completion of behavioral experiments, RNA was extracted from L3-L5 DRG of knockout mice and their littermate controls using a Qiagen RNeasy Microkit (74004) according to the manufacturer’s protocol. qRT-PCR was carried out with Taqman probes against Dnmt3a (Dnmt3a-Mm00432881_m1), Dnmt3b (Dnmt3b-Mm01240113_m1) and Dnmt1 (Dnmt1-Mm01151063_m1). The geometric mean of housekeeping genes Ywhaz (Ywhaz-Mm01158416_g1), Gapdh (Gapdh-Mm99999915_g1), and 18S (18S-Hs99999901_s1) was used to calculate the 2^−ΔCT^ values plotted in **Figure 1**.

### Immunofluorescence

Adult or P2 AdvCre^ERT2^-Dnmt3a^fl/fl^/Dnmt3b^fl/fl^ and their Cre-negative littermates were dosed with tamoxifen (3 × 75 mg/kg *i.p.* in adults as described above or 1x 75 mg/kg *i.p.* in P2 pups). A week (in the case of adults) or 14 days later (at P16), mice were culled by transcardial perfusion with 0.1 M PB and 4% paraformaldehyde. DRG were cryoprotected in 25% sucrose and cut into 10 micron sections on a cryostat. For immunofluorescent staining, sections were incubated for 2 h in blocking serum (10% donkey serum; 0.2% Triton; 0.1% sodium azide in PBS) and stained with antibodies against Dnmt3a (rabbit anti-mouse, 1:1000, Abcam, ab2850 or mouse anti-mouse, 1:500, Santa Cruz, sc-373905), NF200 (chicken anti-mouse, 1:500, Millipore, AB5539) and Iba1 (rabbit anti-mouse, 1:500, Wako, 019-19741) overnight. After three washes in PBS, sections were incubated with secondary antibodies for 2 h at room temperature: Alexa Fluor488 (donkey anti-rabbit or donkey anti-mouse, 1:1000, Invitrogen, A-21206 or A-21202), Alexa Fluor546 (goat anti-chicken, 1:1000, Invitrogen, A-11040), and Alexa Fluor647 (goat anti-rabbit, 1:1000, A-21244). After further washes in PBS, sections were mounted using Fluoromount-G mounting medium with DAPI (Invitrogen) and imaged on a Zeiss LSM 710 confocal microscope.

### Statistical Methods

Behavioral results were evaluated using two-way repeated measures ANOVAs with paw (ipsi vs. contralateral) and time (after CFA or nerve injury) as within-subject variables and treatment or genotype as between-subject variables. For molecular results, we performed independent samples *t*-tests.

## Results

On an RNA level, we were able to demonstrate that administration of tamoxifen to AdvCre^ERT2^-Dnmt3a^fl/fl^/Dnmt3b^fl/fl^ mice resulted in knockdown of Dnmt3a, with no compensatory upregulation of Dnmt1, as measured by qRT-PCR performed on whole DRG (**Figure 1**). Western blots conducted at the time on unperfused mouse DRG also suggested that Dnmt3a was detectable and could be knocked down genetically in AdvCre^ERT2^ positive floxed mice (data not shown).

**FIGURE 2:**
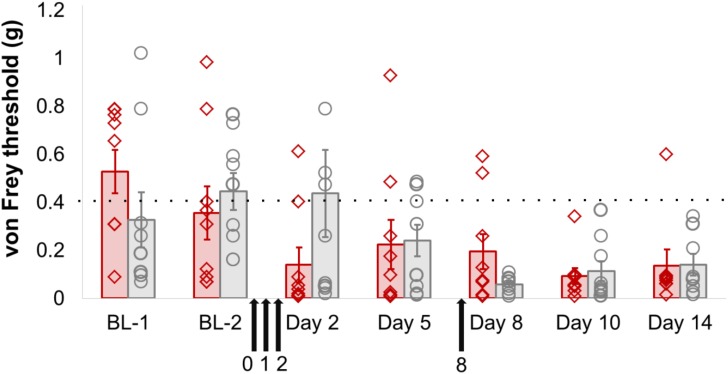
Dnmt3a/3b knockout, even in conjunction with pharmacological Dnmt1 blockade, had no significant effect on mechanical hypersensitivity induced by nerve injury in mice. AdvCre^ERT2^-Dnmt3a^fl/fl^/Dnmt3b^fl/fl^ and their age-matched littermate controls (Dnmt3a^fl/fl^/Dnmt3b^fl/fl^, *n* = 9) were treated with tamoxifen and their mechanical hypersensitivity was assessed using von Frey filaments before (BL-1, BL-2) and after partial sciatic nerve ligation surgery (day 2, day 5, day 8, day 10, day 14). The Dnmt1 inhibitor RG108 was delivered intrathecally 30 min before surgery (day 0), as well as at varying intervals after surgery (day 1, day 2 and day 8, indicated by the black arrows). Plotted are 50% withdrawal thresholds in grams for the ipsilateral paw of each individual animal (diamonds for knockout mice, circles for their littermate controls), as well as their means and standard errors (red and gray bars for knockouts and littermates, respectively). The dotted line indicates the mean baseline withdrawal threshold. See **Supplementary Table 1** for raw data and further details.

However, there did not appear to be any functional consequences of sensory-neuron specific Dnmt3a/3b knockout. Mechanical sensitivity thresholds in mice were unaffected by genotype, either at baseline or after partial sciatic nerve ligation (**Figure 2**, **Supplementary Figure 1** and **Table 1**). A repeated-measures ANOVA with genotype as the dependent variable and time and paw as independent variables revealed a significant main effect of time (pre- and post-surgery, *p* = 0.003), a significant main effect of paw (ipsi- vs. contralateral, *p* = 0.001) and a significant interaction between time × paw, in that the ipsilateral paw showed lowered sensitivity thresholds only after surgery (*p* = 0.02). However, no significant main or interaction effects involving genotype could be observed. These outcomes were not altered by additional pharmacological inhibition of Dnmt1 using intrathecal administration of the small molecule inhibitor RG108 (black arrows). This suggests that the only remaining DNMT in these mice was not responsible for functionally rescuing and therefore camouflaging an effect of Dnmt3a/3b deletion.

The same was true when we measured thermal sensitivity using a hot plate (**Supplementary Figure 2** and **Table 2**): a repeated-measures ANOVA showed a significant main effect of time (baseline vs. day 9 after surgery, *p* = 0.042), but no other significant changes.

**FIGURE 3:**
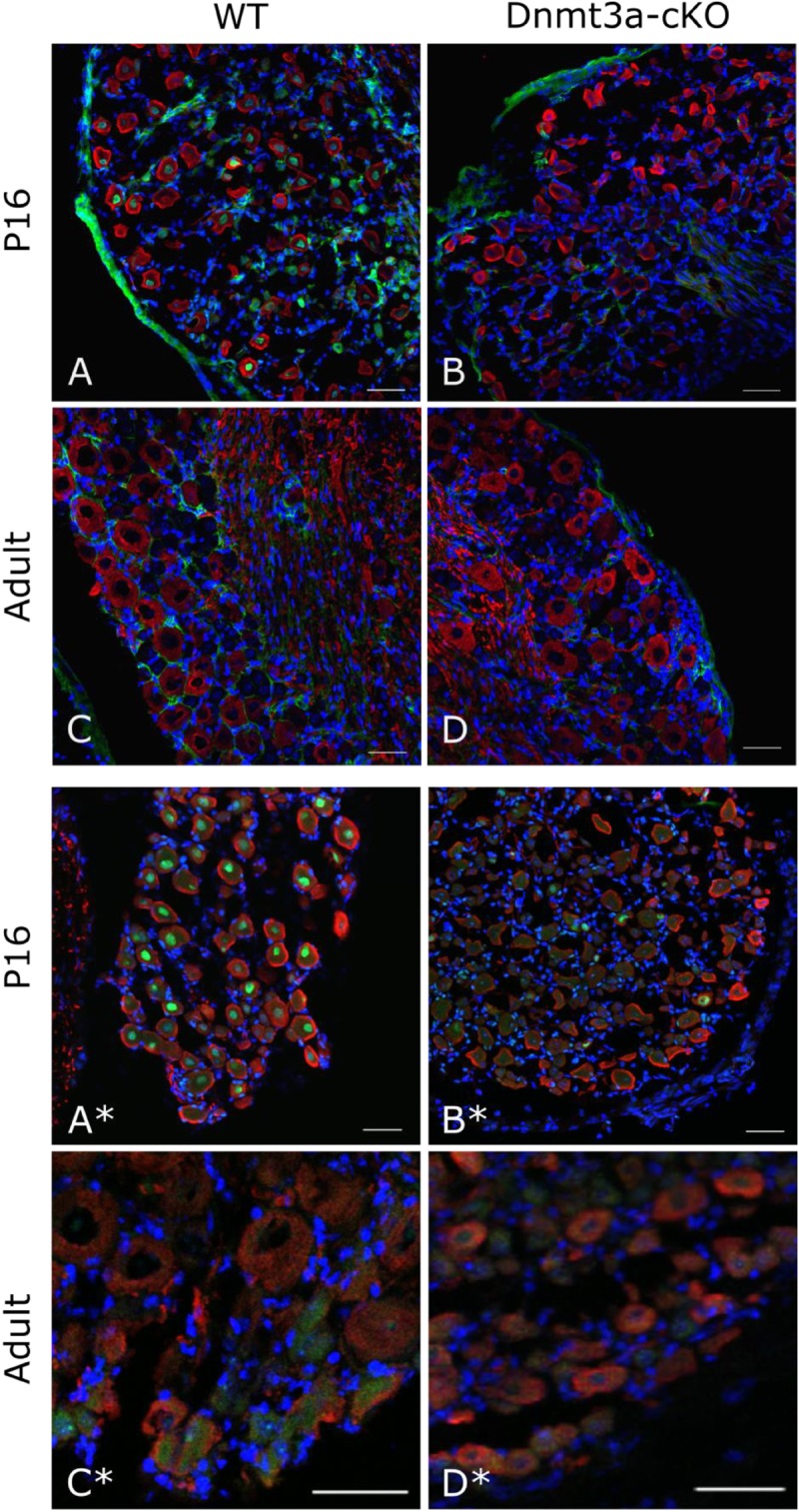
Immunostaining against Dnmt3a indicates lack of neuronal protein expression in the adult. Representative confocal images of DRG cryosections from P16 and adult AdvCre^ERT2^ Dnmt3a KO mice and their littermate controls. Sections were stained with NF200 (red), DAPI (blue) and an antibody against Dnmt3a (green) from Santa Cruz **(A–D)** or Abcam **(A^∗^–D^∗^)**. Scale bar: 50 μm.

To make sense of this finding, we went back to examine DNMT expression levels in mouse DRG in a cell-type specific fashion. On an RNA level, sequencing experiments on purified DRG neurons (Thakur et al., 2014; Motti et al., 2017) suggest that Dnmt1 is the most clearly expressed methyltransferase, while Dnmt3b is absent and Dnmt3a is at or just above the limit of detection thresholds (**Supplementary Figure 3A**). In contrast, unperfused intact whole DRG showed higher levels of Dnmt3a, suggesting that the methyltransferase may be present in non-neuronal tissues or blood. Besides naïve expression levels, we also examined neuronal DNMT levels after nerve injury and found Dnmt1, 3a and 3b mRNA expression to be unchanged (after sciatic nerve crush, **Supplementary Figure 3B** and after partial sciatic nerve ligation, **Supplementary Figures 3C,D**). Finally, immunohistochemistry experiments with two different antibodies indicated that Dnmt3a protein is expressed in DRG neurons during postnatal development (P16) in wild-type but not knockout tissue, yet ultimately downregulated below detection levels in adult mice (**Figure 3**).

**FIGURE 4:**
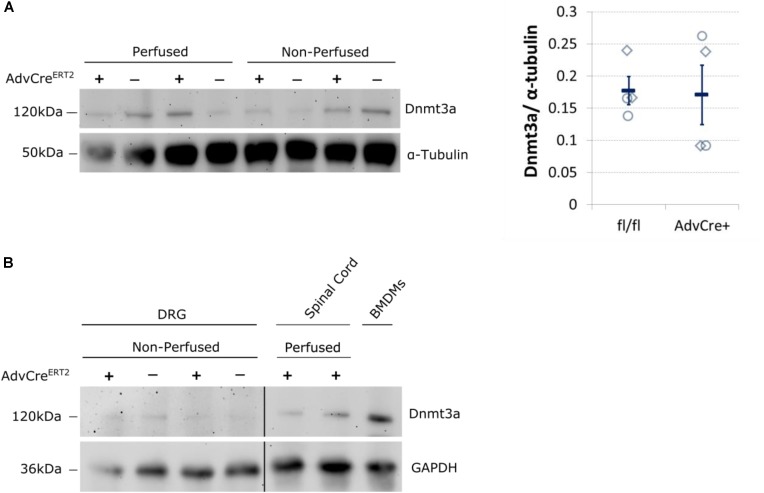
Dnmt3a signal in Western blots does not vary with blood content and is strongest in mouse bone-marrow derived macrophages. **(A)** Dnmt3a signal did not vary with blood content or genotype. Adult DRG were taken from PBS perfused and non-perfused AdvCre^ERT2^ mice (+) and their Cre-negative littermate controls (−). The signal is quantified relative to the housekeeper (α-tubulin). Each data point (diamonds for perfused, circles for non-perfused tissue) represents one biological replicate. Means and their standard errors are also displayed. **(B)** Bone marrow derived macrophages (BMDMs) were cultured from a C57BL/6J mouse for comparison. Samples of spinal cord are also displayed. GAPDH expression used as a protein loading control. Break indicates splicing of blot to remove additional DRG and brain samples.

We therefore think it likely that Dnmt3a protein content in unperfused whole DRG derives from non-neuronal cells. Expression data derived from BLUEPRINT suggest that peripheral blood mononuclear cells (PBMCs) and leukocytes could be possible candidates (**Supplementary Figure 4**). We conducted a few small scale experiments to test these options. PBS perfusion of our tissue did not alter the level of Dnmt3a in Western blots significantly (**Figure 4A**), suggesting that PBMCs are not a likely source of this protein in mouse DRG. Instead, we found that mouse bone-marrow derived macrophages test positively for Dnmt3a in Western blots, making it possible that myeloid cell types express Dnmt3a in adulthood (**Figure 4B**).

**FIGURE 5:**
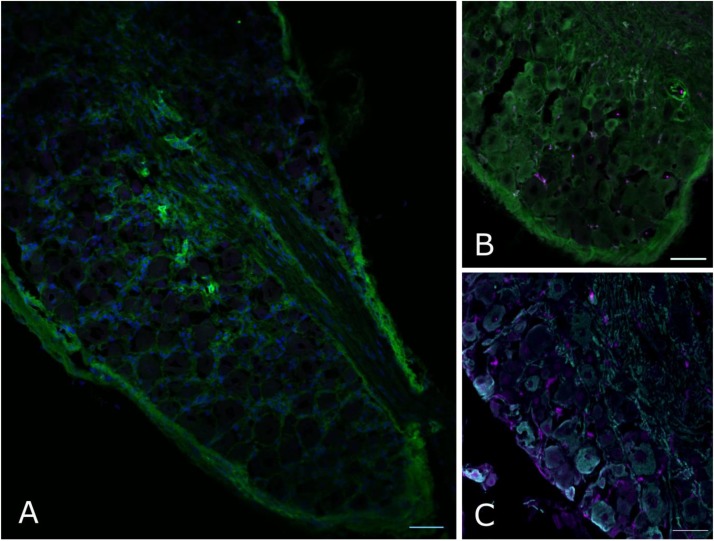
Non-neuronal Dnmt3a signal in adult mouse DRG does not overlap with Iba1 staining. Confocal images of adult mouse DRG cryosections after staining with Dnmt3a (green) and Iba1 (purple) indicate that non-neuronal Dnmt3a signal does not co-localize with Iba1 **(A,B)**. **(C)** Co-staining of NF200 (cyan) and Iba1 (purple). Blue channel: DAPI.

However, when we went on to test non-neuronal Dnmt3a expression with immunofluorescent staining of DRG, a more complex picture emerged: of the two Dnmt3a antibodies we knock-out validated, only the Santa Cruz version produced any signal in adult non-neuronal cell types in our sections (compare **Figures 3A,C** to **A^∗^,C^∗^** and see **Figure 5A**). But this non-neuronal staining did not appear to overlap with Iba1, a marker for myeloid cell types (**Figures 5B,C**). In conclusion then, much more detailed work is needed to ascertain whether Dnmt3a is really present in non-neuronal cells in the adult and, if so, which ones. It is for instance possible that DRG macrophages start expressing Dnmt3a when they adopt certain phenotypes in injury states. Equally, it is possible that the signal observed with the Santa Cruz antibody derives from dividing fibroblasts or satellite glia.

**FIGURE 6:**
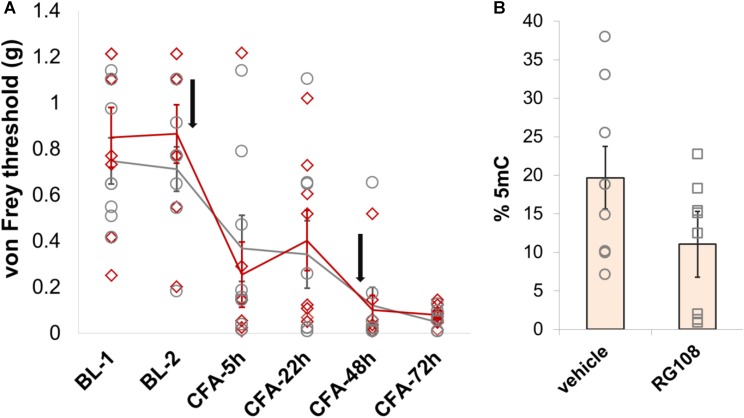
Dnmt1 inhibition has no effect on hypersensitivity induced by inflammation in mice. **(A)** Intrathecal administration of the Dnmt1 inhibitor RG108 30 min before and 47 h after intraplantar CFA administration (black arrows) had no effect on mechanical sensitivity as measured by von Frey. Plotted are 50% withdrawal thresholds in grams for each individual animal (diamonds for RG108 treated mice, circles for their vehicle-treated controls), as well as their means and standard errors (red and gray lines for knockouts and littermates, respectively). See **Supplementary Table 3** for raw data and further details. **(B)** ELISA against global 5-mC levels demonstrated a 43% reduction in DRG methylation in RG108- versus vehicle-treated mice. This effect was near-significant at *p* = 0.055 (one-tailed independent samples *t*-test, with unequal variances assumed).

Finally, given that Dnmt1 appears to be the only DNMT that is expressed in adult neurons to significant levels, we chose to explore its role in nociception in a little more detail. Having observed no behavioral effects of the Dnmt1 inhibitor RG108 in a model of partial sciatic nerve ligation, we chose to further examine Dnmt1 in a model of inflammatory hypersensitivity. RG108 was delivered intrathecally to C57BL/6J wild-type mice 30 min before and 47 h after intraplantar injection of CFA. As was the case after nerve injury, no significant changes in mechanical sensitivity thresholds between groups were measurable (**Figure 6A** and **Supplementary Table 3**), despite a 43% reduction in global 5-mC DNA methylation levels being observed in the dorsal root ganglia of treated mice compared to their vehicle counterparts (**Figure 6B**).

## Discussion

Our results, together with findings from publicly available sequencing data, make us confident that Dnmt3a and Dnmt3b proteins are not expressed at easily detectable level in adult mouse DRG neurons. At mRNA level, Dnmt3b is also completely absent, while low quantities of Dnmt3a can be measured and further reduced via genetic knockout in AdvCre^ERT2^-Dnmt3a^fl/fl^/Dnmt3b^fl/fl^ mice. It is unclear whether this Dnmt3a message has any functional role, given that it is not translated into appreciable amounts of protein in adult wild-type animals. During development, Dnmt3a protein is present at least until P16, and indeed, single cell data suggest it could be found in neurons, particularly non-peptidergic ones, beyond that time: at P20-P23, Dnmt3a mRNA appears to exceed Dnmt1 mRNA levels in single cells (Zeisel et al., 2018).

Upon nerve injury, neuronal mRNA expression of any of the three DNMTs does not appear to be significantly altered in nociceptors. It consequently comes as no surprise that interfering with Dnmt3a and 3b function in sensory neurons has no impact on sensory perception, neither at baseline nor after partial sciatic nerve ligation.

It is not unusual for mRNA to be present in the absence of protein. On a genome-wide level, mRNA abundance has classically been reported to be only moderately predictive of protein levels, with correlations ranging from 30 to 50% (Csardi et al., 2015; Edfors et al., 2016). At steady state, the extent of this disconnect may appear exaggerated due to technical factors, such as failure to correctly take into account missing values, noise and the non-linear relationship between mRNA and proteins (Li et al., 2014; Csardi et al., 2015). Indeed, when re-evaluated with these features in mind, estimated correlations between mRNA and protein content range from a minimum of 56% to a maximum of 85%. Nevertheless, especially in the case of lowly expressed genes like Dnmt3a, it is likely common for mRNA to be degraded or translated at very low rates.

Our findings on DNMT mRNA expression are in keeping with publicly available RNA-seq data on whole human DRG (Flegel et al., 2015; Ray et al., 2018), which report normalized expression values that are similar to those we report here in mouse neurons: clear expression of Dnmt1, borderline expression of Dnmt3a and absence of Dnmt3b message. In contrast, these mRNA data and our other findings run counter to two previously published manuscripts from the same group which present evidence for the involvement of Dnmt3a in nociception in rats and mice (Sun et al., 2017; Zhao et al., 2017). At present, we cannot explain this discrepancy. We used the Santa Cruz antibody reported by Tao and his colleagues, as well as another Abcam antibody, but with neither could replicate the staining patterns observed by the authors in their two papers.

Besides Dnmt3a/3b, our experiments suggest that Dnmt1 may also not be relevant in the context nociception, although the conclusions we can draw are less firm. In neuropathic pain, we temporally inhibited Dnmt1 function using intrathecal delivery of the small molecule inhibitor RG108 30 min before sciatic nerve ligation surgery. We also measured behavioral effects 2 and 8 days after injury, each time within hours of a further injection. At the time we conducted our behavioral experiments, the half-life of RG108 had only been measured *in vitro*, where it was reported to be approximately 20 days (Brueckner et al., 2005). Today, an *in vivo* study suggests that RG108 delivered subcutaneously may only be active for about 4 h (Schneeberger et al., 2016). In our experimental context, its effects were measurable for slightly longer than that, with reduced 5-mC levels still being observed approximately 24 h after the last intrathecal injection (**Figure 6B**). Nevertheless, we have to assume that the drug would not have remained in the system of our mice for much longer than that. And while we observed a drop in global DNA methylation, it is still conceivable that the function of Dnmt1 was not sufficiently inhibited to unmask a phenotype. It has been suggested (Feng et al., 2010) that Dnmt1 has the capacity to act as a *de novo* methyltransferase in neurons, in addition to its main role in maintaining existing DNA methylation patterns. In principle therefore, the enzyme could impact how sensory neuron DNA is methylated in adult animals. Further experiments will be required to resolve the role of Dnmt1 in nociception more unambiguously.

We think that a couple of lessons can be garnered from this evidence that go beyond DNMT function and nociception. Firstly, we proceeded to our behavioral experiments based on qRT-PCR and a Western blot result that appeared to demonstrate knockdown in *n* = 3 animals. However, as can be easily observed from the Western blot ultimately published in this paper: larger scale and more thorough investigations revealed that the protein signal did not actually vary consistently between knockout and wild-type animals (**Figure 4A**). This result is in keeping with our immunohistochemistry data suggesting that Dnmt3a is not expressed in adult sensory neurons and consequently unaffected by Advillin-Cre driven knockout. Moreover, it can serve as an important reminder that small n numbers (< 5) are likely to yield many false positive results (Curtis et al., 2018), especially in the context of mixed tissue where variability between samples can be exacerbated by differences in tissue composition, such as a few extra drops of blood or a few millimeters of additional dorsal root.

Secondly, particularly when studying epigenetics and epigenetic modifiers such as DNMTs, it is vital to conduct studies in a cell-type specific manner. While there are instances in which epigenetic marks across different tissues are correlated (Bakulski et al., 2016), evidence accumulating over the last decade has made it plain that – as a rule – both DNA methylation and histone modifications are highly dependent on cell-type (Martens and Stunnenberg, 2013; Schultz et al., 2015; Halder et al., 2016). This is an unsurprising fact when one considers that it is a cell’s epigenetic profile which determines which genes are accessible and therefore what phenotype the cell adopts once development is complete (Cantone and Fisher, 2013; Heinz et al., 2015).

In the context of pain, this need for cell-type specificity is particularly acute. Most of the models commonly used in the field, be they models of nerve injury (e.g., sciatic nerve ligation, chronic constriction injury, spared nerve injury) or inflammation (e.g., injections of carrageenan or CFA), majorly disrupt cell composition in many areas. For instance, peripheral immune cells infiltrate into nerve or skin, and central nervous system immune cells proliferate in neuropathic models. Consequently, “painful” and “non-painful” experimental groups examined on a global, tissue-unspecific level, will differ vastly in terms of their epigenetic profile, mRNA level and protein content. An apparent pain-related epigenetic change in gene X can therefore sometimes simply reflect the native epigenetic state of an infiltrating immune cell. And consequently, failure to eliminate tissue composition as a confounding factor may lead to a confused picture of the role of epigenetics in chronic pain (Laumet et al., 2015; Sun et al., 2017; Zhao et al., 2017).

## Conclusion

Our study does not currently support a role for DNMTs in sensory neuron processing. In particular, Dnmt3a and Dnmt3b do not appear to be expressed in adult primary sensory neurons at protein level and consequently their knockout in this cell type has no effect on nociception either in naïve animals or in the context of nerve injury.

## Author Contributions

JS and ZH have contributed equally to this MS. JS and ZH carried out the staining and Western blot experiments. FD carried out the behavioral studies. CG performed the intrathecal injections. FD wrote the manuscript, and all other authors read and contributed to its content.

## Conflict of Interest Statement

The authors declare that the research was conducted in the absence of any commercial or financial relationships that could be construed as a potential conflict of interest.

## Supplementary Material

FIGURE S1 Dnmt3a/3b knockout had no significant effect on mechanical sensitivity in the paw contralateral to nerve injury. AdvCreERT2-Dnmt3afl/fl/ Dnmt3bfl/fl and their age-matched littermate controls (Dnmt3afl/fl/Dnmt3bfl/fl, *n* = 9) were treated with tamoxifen and their mechanical hypersensitivity was assessed using von Frey filaments before (BL-1, BL-2) and after partial sciatic nerve ligation surgery (day 2, day 5, day 8, day 10, day 14). The Dnmt1 inhibitor RG108 was delivered intrathecally 30 min before surgery (day 0), as well as at varying intervals after surgery (day 1, day 2 and day 8, indicated by the black arrows). Plotted are 50% withdrawal thresholds in grams for the contralateral paw of each individual animal (diamonds for knockout mice, circles for their littermate controls), as well as their means and standard errors (red and gray bars for knockouts and littermates, respectively). The dotted line indicates the mean baseline withdrawal threshold. See **Supplementary Table 1** for raw data and further details.

FIGURE S2 Dnmt3a/3b knockout had no effect on thermal sensitivity. AdvCreERT2-Dnmt3afl/fl/Dnmt3bfl/fl and their age-matched littermate controls (Dnmt3afl/fl/Dnmt3bfl/fl, *n* = 9) were treated with tamoxifen and their thermal hypersensitivity was assessed using a hotplate test before (BL) and nine days after partial sciatic nerve ligation surgery (day 9). Plotted is the temperature (in °C) at which individual mice displayed pain (flicking or licking of the paw) or escape behavior (jumping): diamonds for knockout mice, circles for their littermate controls. Means and standard errors are also displayed (red and gray lines for knockout and littermate controls, respectively). See **Supplementary Table 2** for raw data and further details.

FIGURE S3 RNA expression level of DNMTs in purified DRG neurons. **(A)** DNMT expression levels in whole DRG, dissociated DRG and magnetically sorted nociceptors, derived from publicly available data (Thakur et al., 2014). The dotted line indicates the expression threshold suggested by the authors as appropriate for their dataset. **(B)** DNMT expression levels in sorted sensory neurons after sciatic nerve crush, derived from publicly available data (Motti et al., 2017). **(C,D)** DNMT expression levels in magnetically sorted nociceptors after partial sciatic nerve ligation in **(C)** male and **(D)** female mice. Expression values are displayed as a fraction of a housekeeping gene. Circles indicate individual biological replicates. Their means and standard errors are also plotted.

FIGURE S4 mRNA expression levels of Dnmt3a in various blood-derived cell populations as derived from BLUEPRINT. Dnmt3a message is present in human neutrophils, macrophages, inflammatory macrophages, alternatively activated macrophages, monocytes, dendritic cells, various different kinds of T cells, naïve B cells and peripheral blood-derived mononuclear cells (PBMCs). Data are expressed in FPKM and derived from the BLUEPRINT (Martens and Stunnenberg, 2013) website (data release 2016-08).

TABLE S1 No effect of Dnmt3a/3b knockout on nerve injury: von Frey data.

TABLE S2 No effect of Dnmt3a/3b knockout on nerve injury: hot plate data.

TABLE S3 No effect of Dnmt1 inhibition on inflammatory pain: von Frey data.
