# Supplementary material for: Negative Evidence for a Functional Role of Neuronal DNMT3a in Persistent Pain
Source: Front Mol Neurosci. 2018 Sep 12;11:332. doi: 10.3389/fnmol.2018.00332 (PMC6143791; doi:10.3389/fnmol.2018.00332)
Supplement: FIGURE S1 — Dnmt3a/3b knockout had no significant effect on mechanical sensitivity in the paw contralateral to nerve injury. AdvCreERT2-Dnmt3afl/fl/ Dnmt3bfl/fl and their age-matched littermate controls (Dnmt3afl/fl/Dnmt3bfl/fl, n = 9) were treated with tamoxifen and their mechanical hypersensitivity was assessed using von Frey filaments before (BL-1, BL-2) and after partial sciatic nerve ligation surgery (day 2, day 5, day 8, day 10, day 14). The Dnmt1 inhibitor RG108 was delivered intrathecally 30 min before surgery (day 0), as well as at varying intervals after surgery (day 1, day 2 and day 8, indicated by the black arrows). Plotted are 50% withdrawal thresholds in grams for the contralateral paw of each individual animal (diamonds for knockout mice, circles for their littermate controls), as well as their means and standard errors (red and gray bars for knockouts and littermates, respectively). The dotted line indicates the mean baseline withdrawal threshold. See Supplementary Table 1 for raw data and further details. [file Presentation_1.pdf]

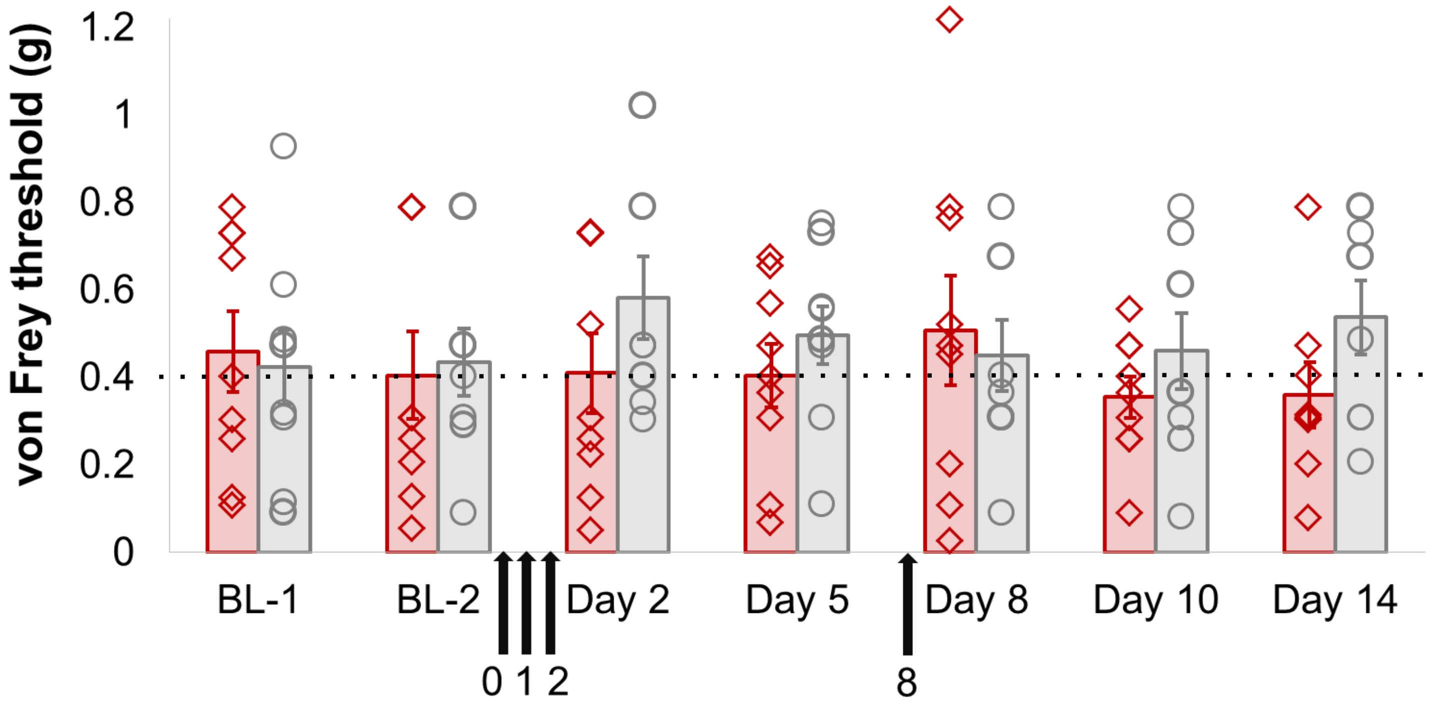

Suppl. Fig 1. Dnmt3a/3b knockout had no significant effect on mechanical sensitivity in the paw contralateral to nerve injury
AdvCreERT2-Dnmt3afl/fl/Dnmt3bfl/fl and their age-matched littermate controls (Dnmt3afl/fl/Dnmt3bfl/fl, n = 9) were treated with tamoxifen and their mechanical hypersensitivity was assessed using von Frey filaments before (BL-1, BL-2) and after partial sciatic nerve ligation surgery (Day 2, Day 5, Day 8, Day 10, Day 14). The Dnmt1 inhibitor RG108 was delivered intrathecally 30min before surgery (day 0), as well as at varying intervals after surgery (day 1, day 2 and day 8, indicated by the black arrows). Plotted are 50% withdrawal thresholds in grams for the contralateral paw of each individual animal (diamonds for knockout mice, circles for their littermate controls), as well as their means and standard errors (red and grey bars for knockouts and littermates, respectively). The dotted line indicates the mean baseline withdrawal threshold. See Suppl. Table 1 for raw data & further details.

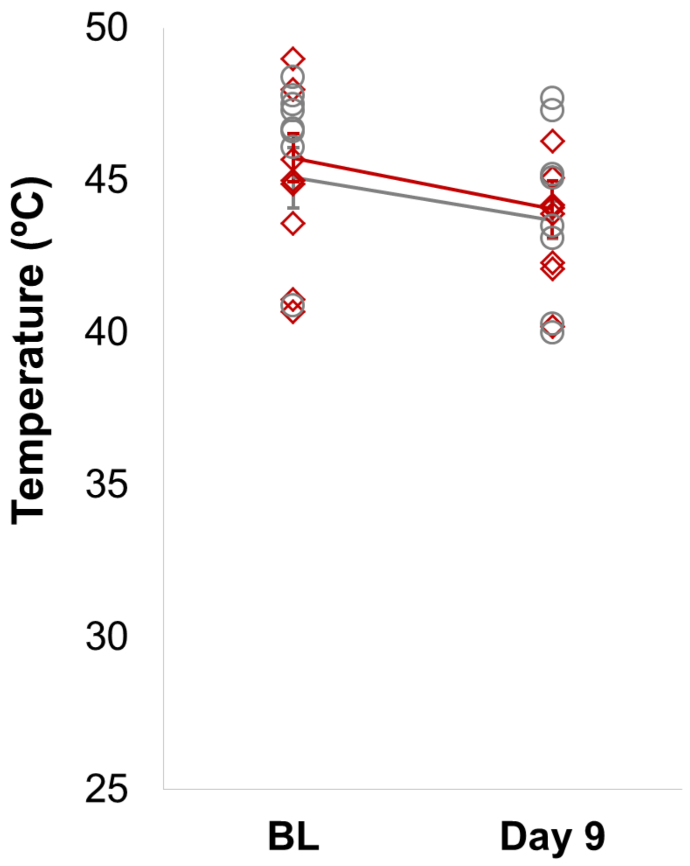

Suppl. Fig 2. Dnmt3a/3b knockout had no effect on thermal sensitivity
AdvCreERT2-Dnmt3afl/fl/Dnmt3bfl/fl and their age-matched littermate controls (Dnmt3afl/fl/Dnmt3bfl/fl, n = 9) were treated with tamoxifen and their thermal hypersensitivity was assessed using a hotplate test before (BL) and nine days after partial sciatic nerve ligation surgery (Day 9). Plotted is the temperature (in °C) at which individual mice displayed pain (flicking or licking of the paw) or escape behaviour (jumping): diamonds for knockout mice, circles for their littermate controls. Means and standard errors are also displayed (red and grey lines for knockout and littermate controls, respectively). See Suppl. Table 2 for raw data & further details.

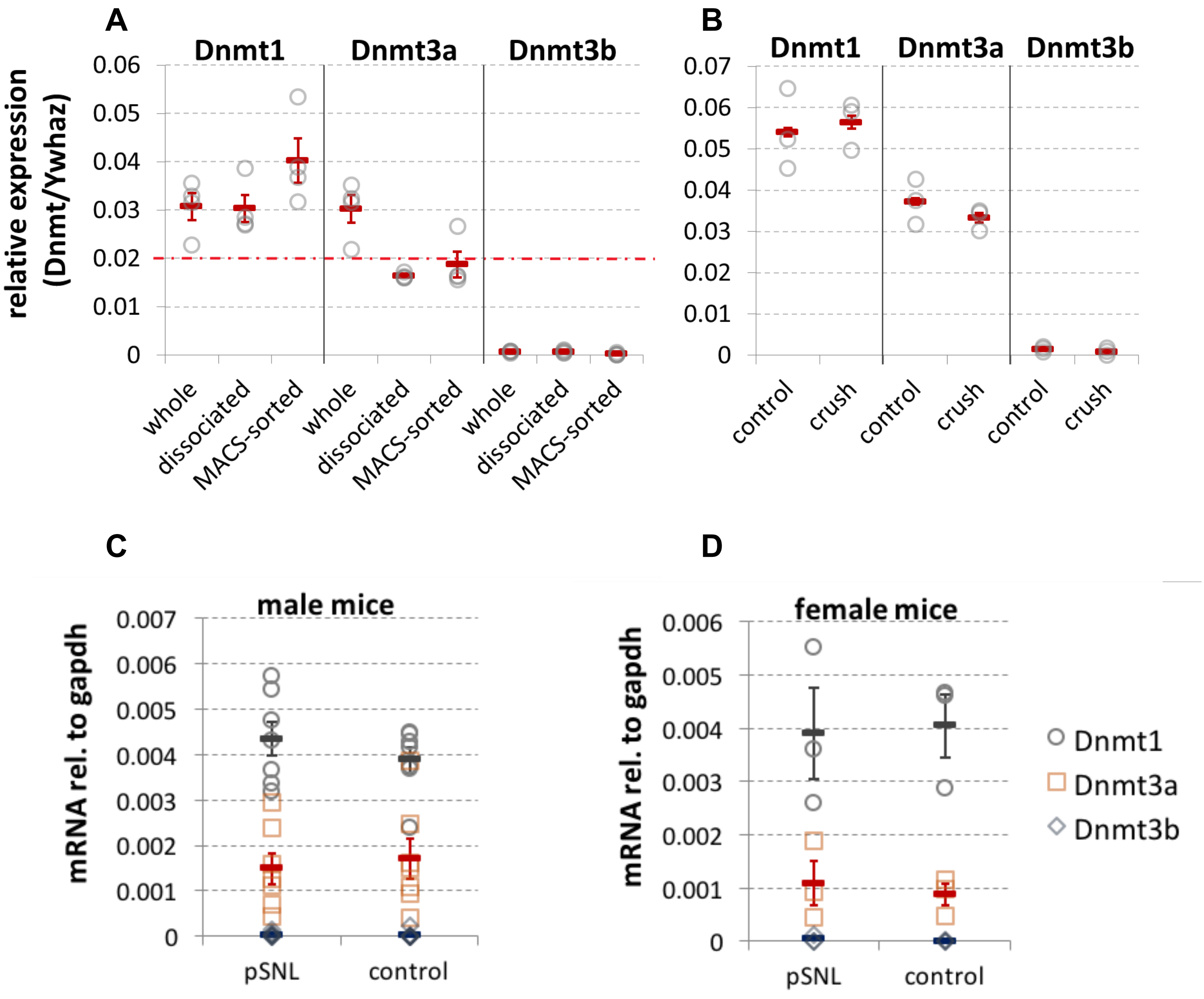

Suppl. Fig 3. RNA expression level of DNMTs in purified DRG neurons

A, DNMT expression levels in whole DRG, dissociated DRG and magnetically sorted nociceptors, derived from publicly available data (Thakur et al., 2014). The dotted line indicates the expression threshold suggested by the authors as appropriate for their dataset.

B, DNMT expression levels in sorted sensory neurons after sciatic nerve crush, derived from publicly available data (Motti et al., 2017).

C & D, DNMT expression levels in magnetically sorted nociceptors after partial sciatic nerve ligation in C, male and D, female mice.

Expression values are displayed as a fraction of a housekeeping gene. Datapoints indicate individual biological replicates. Their means and standard errors are also plotted.

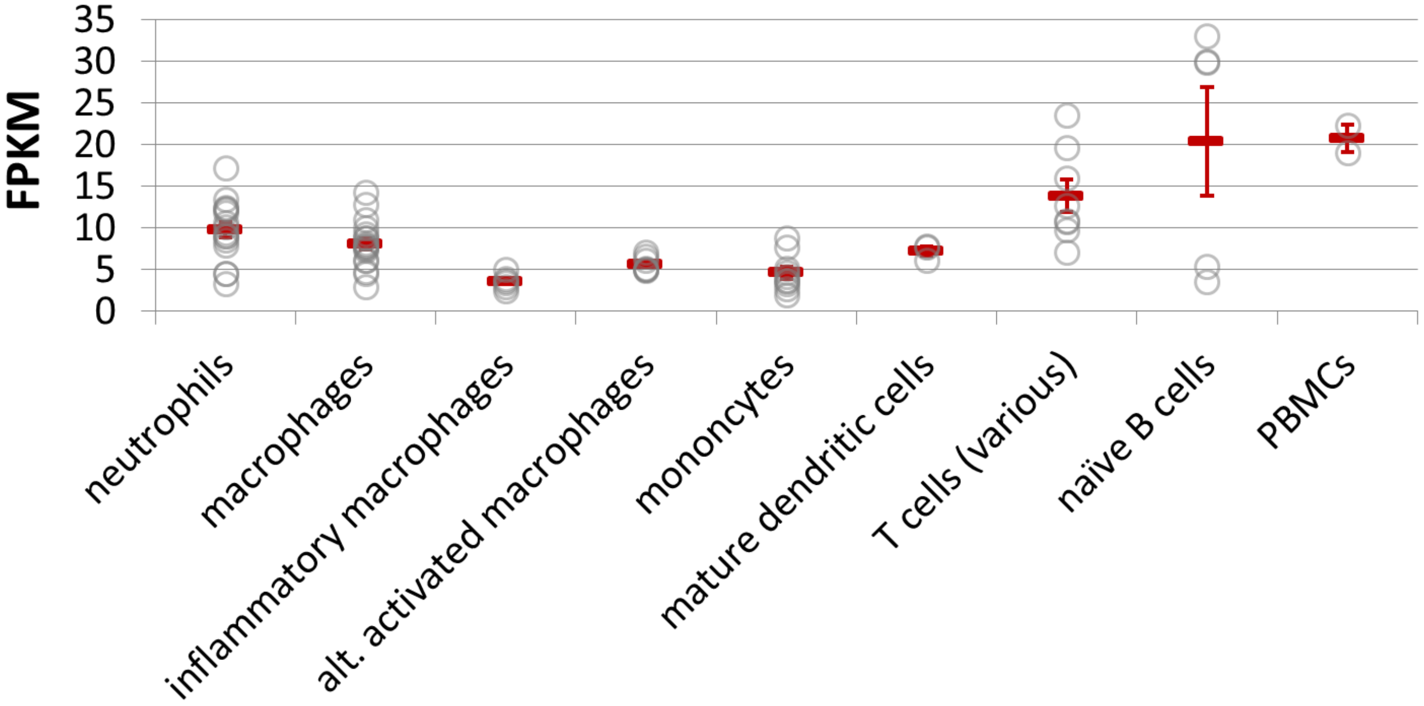

Suppl. Fig 4. mRNA expression levels of Dnmt3a in various blood-derived cell populations - data taken from BLUEPRINT
Dnmt3a message is present in human neutrophils, macrophages, inflammatory macrophages, alternatively activated macrophages, monocytes, dendritic cells, various different kinds of T cells, naïve B cells and peripheral blood-derived mononuclear cells (PBMCs). Data are expressed in FPKM and derived from the BLUEPRINT website (data release 2016-08, Martens and Stunnenberg, 2013).
